# A Novel Necroptosis-Related lncRNA Signature Predicts the Prognosis of Lung Adenocarcinoma

**DOI:** 10.3389/fgene.2022.862741

**Published:** 2022-03-17

**Authors:** Yinliang Lu, XueHui Luo, Qi Wang, Jie Chen, Xinyue Zhang, YueSen Li, Yuetong Chen, Xinyue Li, Suxia Han

**Affiliations:** Department of Radiation Oncology, First Affiliated Hospital of Xi’an Jiaotong University, Xi’an, China

**Keywords:** lung adenocarcinoma, necroptosis gene, long noncoding RNA, tumor immune microenvironment, prognostic signature

## Abstract

**Background:** Necroptosis is closely related to the tumorigenesis and development of cancer. An increasing number of studies have demonstrated that targeting necroptosis could be a novel treatment strategy for cancer. However, the predictive potential of necroptosis-related long noncoding RNAs (lncRNAs) in lung adenocarcinoma (LUAD) still needs to be clarified. This study aimed to construct a prognostic signature based on necroptosis-related lncRNAs to predict the prognosis of LUAD.

**Methods:** We downloaded RNA sequencing data from The Cancer Genome Atlas database. Co-expression network analysis, univariate Cox regression, and least absolute shrinkage and selection operator were adopted to identify necroptosis-related prognostic lncRNAs. We constructed the predictive signature by multivariate Cox regression. Kaplan–Meier analysis, time-dependent receiver operating characteristics, nomogram, and calibration curves were used to validate and evaluate the signature. Subsequently, we used gene set enrichment analysis (GSEA) and single-sample gene set enrichment analysis (ssGSEA) to explore the relationship between the predictive signature and tumor immune microenvironment of risk groups. Finally, the correlation between the predictive signature and immune checkpoint expression of LUAD patients was also analyzed.

**Results:** We constructed a signature composed of 7 necroptosis-related lncRNAs (AC026355.2, AC099850.3, AF131215.5, UST-AS2, ARHGAP26-AS1, FAM83A-AS1, and AC010999.2). The signature could serve as an independent predictor for LUAD patients. Compared with clinicopathological variables, the necroptosis-related lncRNA signature has a higher diagnostic efficiency, with the area under the receiver operating characteristic curve being 0.723. Meanwhile, when patients were stratified according to different clinicopathological variables, the overall survival of patients in the high-risk group was shorter than that of those in the low-risk group. GSEA showed that tumor- and immune-related pathways were mainly enriched in the low-risk group. ssGSEA further confirmed that the predictive signature was significantly related to the immune status of LUAD patients. The immune checkpoint analysis displayed that low-risk patients had a higher immune checkpoint expression, such as CTLA-4, HAVCR2, PD-1, and TIGIT. This suggested that immunological function is more active in the low-risk group LUAD patients who might benefit from checkpoint blockade immunotherapies.

**Conclusion:** The predictive signature can independently predict the prognosis of LUAD, helps elucidate the mechanism of necroptosis-related lncRNAs in LUAD, and provides immunotherapy guidance for patients with LUAD.

## Introduction

Lung cancer is one of the most frequently diagnosed cancers and the leading cause of cancer-related deaths worldwide (Ferlay et al., 2021). Lung cancer is usually divided into non-small cell lung cancer (NSCLC) and small cell lung cancer; 85% of patients are NSCLC, of which lung adenocarcinoma (LUAD) accounts for about 50% (Thai et al., 2021). Recently, substantial improvements, such as chemotherapy, radiotherapy, and immunotherapy, have been made in the treatment of NSCLC patients. However, there is still a proportion of patients with distant metastasis that cannot be effectively treated at an early stage due to the lack of specific biomarkers, resulting in poor 5-year survival rates (Jurisic et al., 2020). Therefore, the identification of a reliable and specific biomarker for diagnosis and prognosis is urgently crucial for NSCLC.

Necroptosis is a form of programmed inflammatory cell death mediated by receptor-interacting protein kinases RIPK1, RIPK3, and mixed lineage kinase domain-like protein (MLKL). Necroptosis is characterized by early loss of plasma membrane integrity, leakage of intracellular contents, and organelle swelling (Krysko et al., 2017; Jiao et al., 2018). Recent studies have indicated that necroptosis has an important role in tumorigenesis, tumor metastasis, and tumoral immune response (Gong et al., 2019). Of note is the fact that necroptosis appears to be antitumorigenic or protumorigenic, depending on the tumor type and conditions during tumorigenesis (Yan et al., 2022). RIPK3 may restrict myeloid leukemogenesis and the differentiation of leukemia-initiating cells by promoting RIPK3–MLKL-mediated necroptosis (Höckendorf et al., 2016). Necroptosis could promote pancreatic cancer cell migration and invasion by the release of CXCL5 (Ando et al., 2020). Necroptosis blockage by MLKL ablation could substantially decrease the lung metastasis of breast cancer cells (Jiao et al., 2018). In addition, necroptosis is expected to develop an inflammatory tumor immune microenvironment *via* releasing damage-associated molecular patterns (DAMPs), cytokines, and/or chemokines in the tumor microenvironment, resulting in tumor-promoting or anti-tumor effects (Sprooten et al., 2020). On one hand, necroptotic tumor cells attract macrophages and DC cells, which are activated by DAMPs and cytokines. The activated DC cells migrate to the lymph nodes and activate naive CD4^+^ and CD8^+^ T cells. The naive T cells are activated and differentiated into effector T cells that leave the lymph nodes, re-enter the blood circulation, and infiltrate into tumor tissue to produce anti-tumor effects (Sancho et al., 2009). RIPK1 expression and NF-κB activation during necroptotic cell death are necessary for efficient cross-priming and antitumor immunity (Yatim et al., 2015). Consistently, vaccination with necroptotic cancer cells could also induce efficient antitumor immunity in an experimental mouse model (Aaes et al., 2016). On the other hand, necroptotic tumor cells also attract myeloid suppressor cells and tumor-associated macrophages, resulting in tumor-associated immunosuppression. Necroptosis-induced CXCL1 promoted pancreatic cancer progression *via* tumor-associated macrophage-induced immune suppression (Seifert et al., 2016). What is mentioned above implies the potential of targeting necroptosis as a novel cancer therapy, especially for immunotherapy.

Long non-coding RNAs (lncRNAs) are non-coding RNAs with transcripts of more than 200 nucleotides. Growing evidence has ascertained that lncRNAs are involved in the progression and metastasis of NSCLC and were associated with the immune pathway (Pang et al., 2021). LINC01748 exerted carcinogenic effects in NSCLC cell lines by regulating the microRNA-520a-5p/HMGA1 axis (Tan et al., 2022). lncRNA-SChLAP1 was verified to induce NSCLC progression and immune evasion by regulating the AUF1/PD-L1 pathway (Du et al., 2021). In addition, several studies demonstrated that lncRNA could also regulate necroptosis *via* functioning as competitive RNAs to influence the expression of target genes. lncRNA-107053293 was demonstrated to regulate necroptosis by acting as a competing endogenous RNA of miR-148a-3p (Wang W et al., 2020). The depletion of Linc00176 disrupted the cell cycle and induced necroptosis in hepatocellular carcinoma *via* regulating the expression of miRNAs, such as miR-9 and miR-185 (Tran et al., 2018). Based on the important role of lncRNA on the tumor, the prognostic signatures based on lncRNAs of LUAD patients have been widely introduced (Chen H et al., 2021; Xu et al., 2021). Nevertheless, research on necroptosis-related lncRNAs (NRlncRNAs) in LUAD prognosis and tumor immune microenvironment (TIME) has not been reported.

In this study, we constructed a novel predictive signature based on NRlncRNAs to forecast the prognosis of LUAD. We also validated its clinical value and confirmed that this signature can be used as a predictor of immunotherapy, which may offer a guiding function for clinicians.

## Materials and Methods

### Preparation of Transcriptomic Data and Clinical Information

We downloaded the transcriptome RNA sequencing data of LUAD samples from The Cancer Genome Atlas (TCGA) (https://portal.gdc.cancer.gov/). Meanwhile, we obtained the corresponding clinical parameters of these patients and excluded patients with missing overall survival (OS) or poor OS (less than 60 days) to reduce statistical bias in this analysis.

### Identification of Necroptosis-Related lncRNA

A list of 67 necroptosis genes was obtained from previously reported literature (Zhao et al., 2021). The correlations between 67 necroptosis-related genes and lncRNA expression were analyzed *via* Pearson correlation analysis. All NRlncRNAs (2,154) should conform to the standard of correlation coefficients (|Pearson R|) >0.4 and *p* <0.001. Then, we obtained 1,061 differentially expressed lncRNAs [log2 fold change > 1, false discovery rate (FDR) <0.05] after screening the synthetic data matrix by Strawberry Perl V-5.30.0 (https://www.perl.org/) and R software V-4.1.2 (https://www.r-project.org/) with limma R package.

### Establishment and Validation of the Risk Signature According to Necroptosis-Related lncRNAs in LUAD

The entire 481 TCGA set of LUAD was divided into a train risk set and a test risk set randomly by the caret R package. The ratio was 1:1. The train set was used to construct a necroptosis-related lncRNA signature, and the test set and entire set were applied to validate the signature. Combined with the clinical information of LUAD in TCGA, we screened and obtained 40 NRlncRNAs linked to OS significantly by univariate Cox (uni-Cox) regression analysis (*p* < 0.05). Subsequently, we performed least absolute shrinkage and selection operator (LASSO) Cox analysis (using the penalty parameter estimated by 10-fold cross-validation) *via* the glmnet R package to screen out optimal lncRNAs associated with LUAD prognosis. This method aims to prevent over-fitting during modeling. Finally, a prognostic risk signature based on the optimal lncRNAs was established with the multivariate Cox (multi-Cox) regression analysis, and the risk score of every patient with LUAD was calculated based on the following formula:

$$\text{risk score}=\overset{\text{n}}{\underset{\text{i}=1}{\sum }}\text{Coef}\left(\text{i}\right)\times \text{Expr}\left(\text{i}\right)$$

Coef(i) and Expr(i) represent the regression coefficient of the multi-Cox regression analysis for each lncRNA and each lncRNA expression level, respectively. The patients were stratified into low- and high-risk groups, with the risk score as the cutoff. Kaplan–Meier method and log-rank test were conducted to analyze whether there is a difference in the OS of LUAD patients between the low- and high-risk groups using the survival R package.

We evaluated the prognostic value of the established risk signature between the model and the clinical characteristics *via* chi-square test. Uni-Cox and multi-Cox regression analyses were performed to explore whether the prognostic signature was a potential independent prognostic indicator for patients with LUAD, and the results were visualized with two forest maps. Several receiver operating characteristic (ROC) curves were generated, and the area under the ROC curve (AUC) was calculated by the survival, survminer, and timeROC R packages to validate the predictive value of the prognostic signature.

### Nomogram and Calibration

We combined the risk score with the clinical variables of age, gender, N stage, T stage, M stage, and tumor stage to set up a nomogram for the 1-, 3-, and 5-year OS of LUAD patients by the rms R package. Correction curves based on the Hosmer–Lemeshow test were applied to illustrate the uniformity between the actual outcome and the signature prediction outcome.

### Enrichment of Functions and Pathways in the Risk Prognosis Signature

We used gene set enrichment analyses (GSEA) software 4.1.2 (http://www.gsea-msigdb.org/gsea/index.jsp) to carry out GSEA and to identify significantly enriched pathways between the low- and high-risk groups. Values of *p* <0.05 and FDR <0.25 were considered the thresholds for statistical significance. The results were visualized by the gridExtra, grid, and ggplot2 R packages.

### Estimation of the Tumor Immune Microenvironment of the Prognostic Signature

To figure out the relationship between this signature and TIME, firstly, we calculated the infiltration values for TCGA-LUAD dataset samples based on 7 algorithms: XCELL (Aran et al., 2017), TIMER (Li T et al., 2017; Li et al., 2020), QUANTISEQ (Finotello et al., 2019), MCPCOUNTER (Dienstmann et al., 2019), EPIC (Racle et al., 2017), CIBERSORT-ABS (Tamminga et al., 2020), and CIBERSORT (Chen et al., 2018). Using Spearman correlation analysis, the relationship of immune cell subpopulations and risk score value was evaluated. Wilcoxon signed-rank test, limma, scales, ggplot2, ggtext, tidyverse, and ggpubr R packages were applied, and the results are displayed in a bubble chart. Then, we explored the abundance of immune cells and stromal cells between different groups. The StromalScore, ImmuneScore, and ESTIMATEScore (StromalScore + ImmuneScore) of each patient were calculated. Their differences were compared using the Wilcoxon signed-rank test, and *p* <0.05 was considered to be significant. Subsequently, single-sample GSEA (ssGSEA) was conducted for scoring LUAD-infiltrating immune cells to quantify their relative content *via* the “GSVA” package. The scores of immune cells and pathways in different groups are shown on multi-boxplots, respectively. Finally, we also made comparisons about the immune checkpoint activation between low- and high-risk groups by the ggpubr R package.

## Results

### Identification of Necroptosis-Related lncRNAs in LUAD Patients

The detailed flow diagram of our study is exhibited in Supplementary Figure S1. The transcriptome data of LUAD downloaded from TCGA included 59 normal samples and 539 tumor samples. We distinguished the mRNAs and lncRNAs by GTF files. According to the expression of 67 necroptosis genes and differentially expressed lncRNAs between normal and tumor samples, we finally obtained 1,016 NRlncRNAs (Supplementary Table S1), including 97 downregulated lncRNAs and 919 upregulated ones (Supplementary Figure S2).

### Construction of the Necroptosis-Related lncRNA Predictive Signature

**FIGURE 1:**
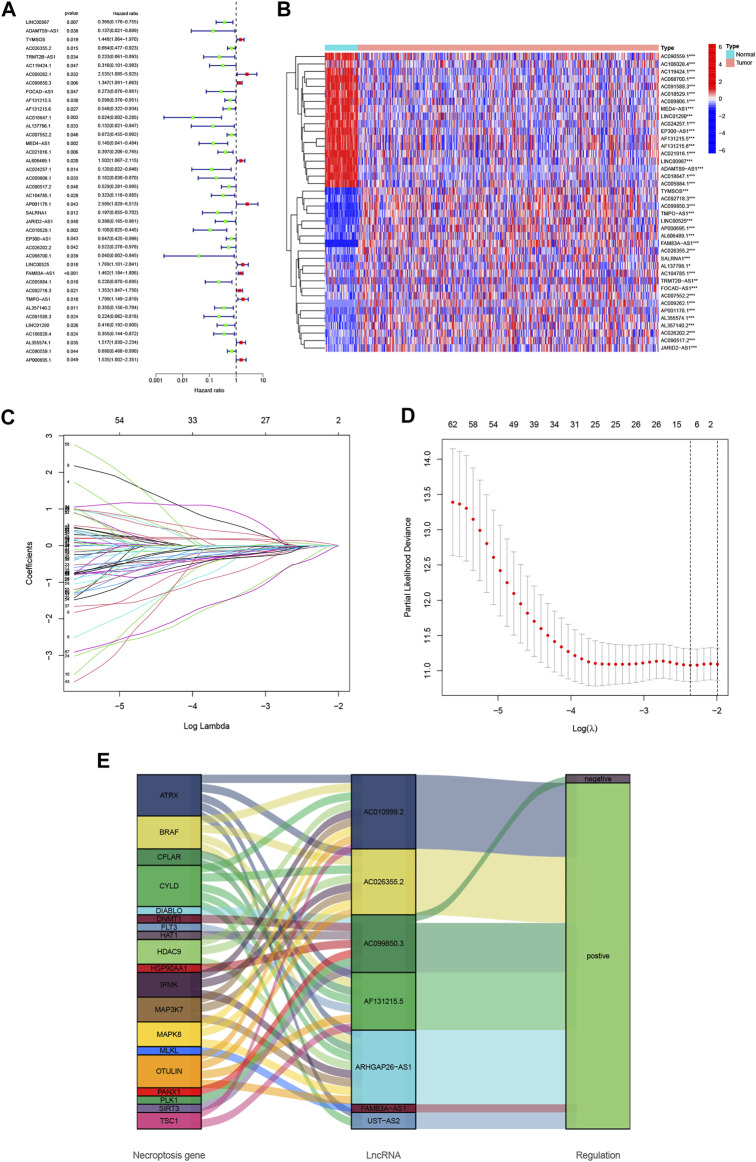
Identification of necroptosis-related lncRNA prognostic signature in lung adenocarcinoma (LUAD). **(A)** Forest plot of 40 necroptosis-related lncRNAs selected by univariate Cox regression analysis. **(B)** The differential expressions of 40 necroptosis-related lncRNAs linked to survival between LUAD and normal samples. **(C)** The 10-fold cross-validation for variable selection in the least absolute shrinkage and selection operator (LASSO) algorithm. **(D)** The LASSO coefficient profile of necroptosis-related lncRNAs. **(E)** The Sankey diagram of the connection between 19 necroptosis genes and 7 necroptosis-related lncRNAs. *p < 0.05, **p < 0.01, ***p < 0.001.

Using uni-Cox regression analysis in the TCGA train set, we obtained 40 NRlncRNAs which were significantly correlated with OS and made a heat map (Figures 1A,B). To avoid overfitting and improve the accuracy and explainability of the prognostic signature, we performed the LASSO-penalized Cox analysis on these lncRNAs and extracted 19 lncRNAs related to necroptosis in LUAD when the first-rank value of Log(λ) was the minimum likelihood of deviance (Figures 1C,D). Subsequently, we constructed the predictive signature composed of 7 NRlncRNAs (AC026355.2, AC099850.3, AF131215.5, UST-AS2, ARHGAP26-AS1, FAM83A-AS1, and AC010999.2) *via* multi-Cox regression analysis. Of those lncRNAs, 6 lncRNAs were regulated positively by necroptosis genes in the Sankey diagram (Figure 1E). Meanwhile, some of those lncRNAs (AC099850.3, AF131215.5, and FAM83A-AS1) were demonstrated to be highly associated with NSCLC previously. Subsequently, the risk score of every LUAD patient was calculated based on correlation coefficients calculated by multivariate Cox regression analysis, and the patients were divided into low- and high-risk groups according to the median value of the risk score. The risk score was calculated as follows: risk score = (−0.3641 × AC026355.2 expression) + (0.1747 × AC099850.3 expression) + (−0.3943 × AF131215.5 expression) + (−0.6257 × UST-AS2 expression) + (−2.8454 × ARHGAP26-AS1 expression) + (0.3281 × FAM83A-AS1 expression) + (−2.1752 × AC010999.2 expression) (Supplementary Table S2).

### Prognosis Values of the Necroptosis-Related lncRNA Signature

**FIGURE 2:**
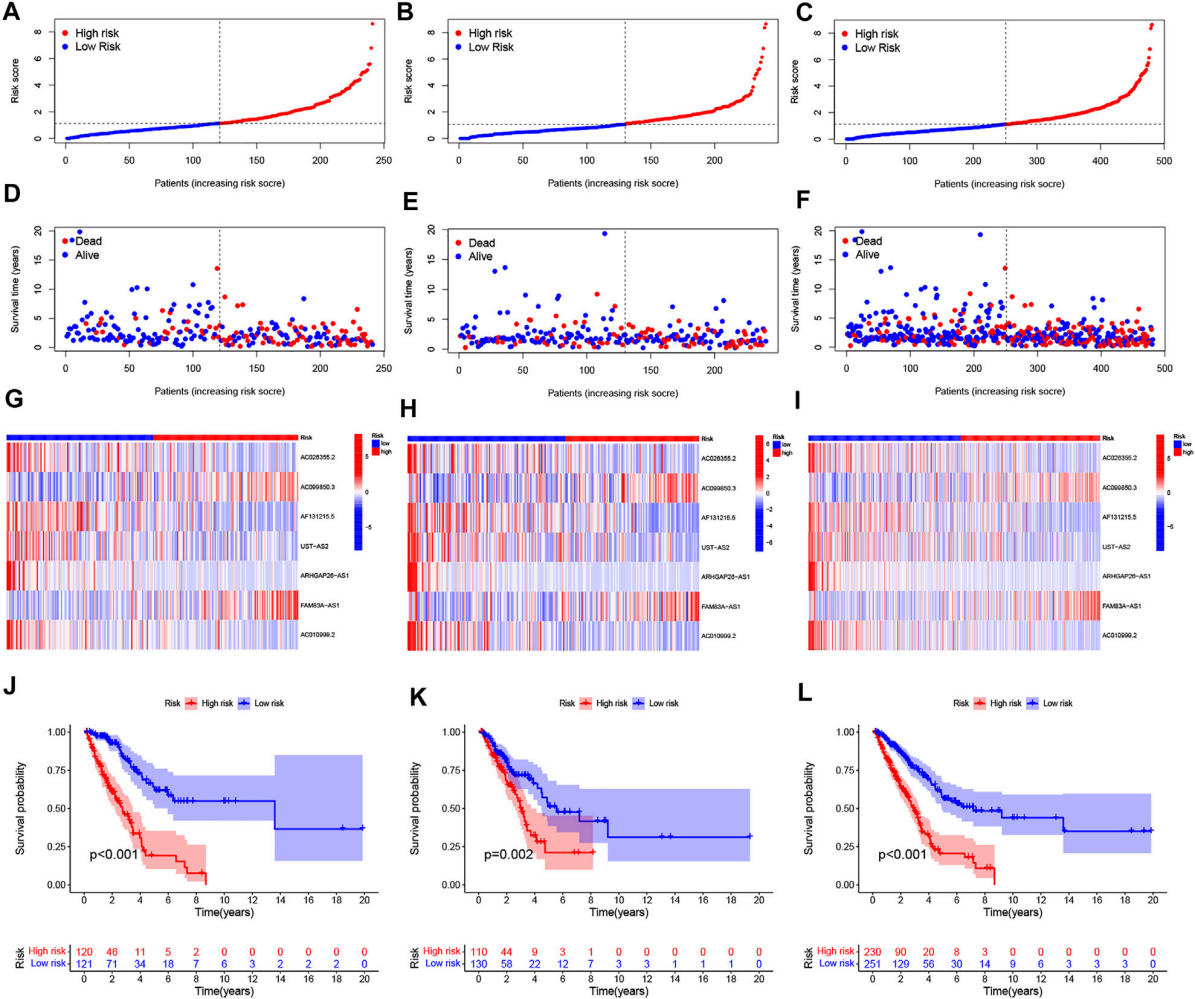
Prognosis values of the 7 necroptosis-related lncRNA signatures in the train, test, and entire sets. The distribution of risk scores **(A–C)**, survival time and survival status **(D–F)**, heat maps of 7 lncRNA expressions **(G–I)**, and Kaplan–Meier survival curves of overall survival of LUAD patients **(J–L)** between low- and high-risk groups in the train, test, and entire sets, respectively.

**FIGURE 3:**
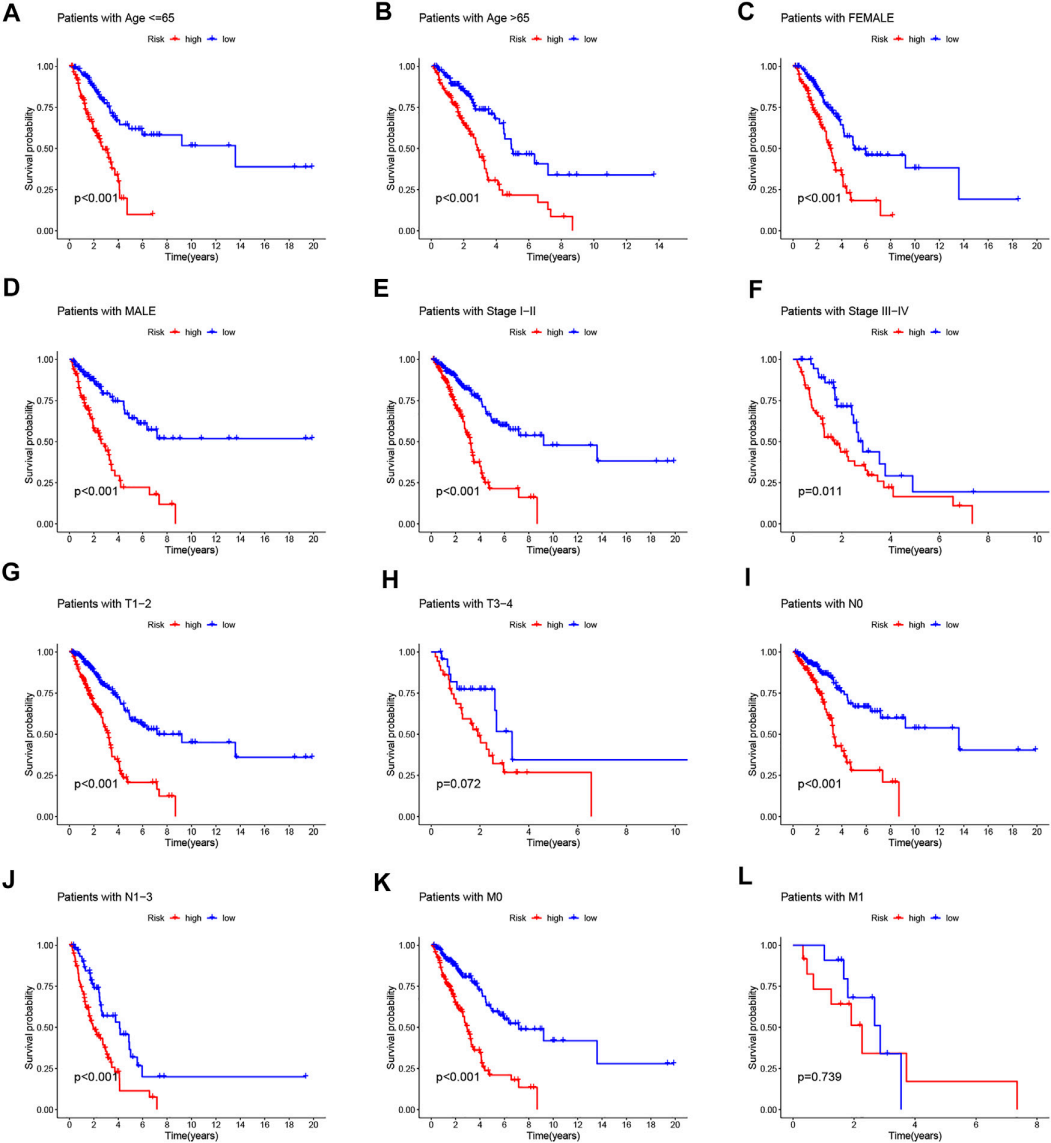
Kaplan–Meier survival curves of low- and high-risk groups sorted by different clinicopathological variables. **(A,B)** Age, **(C,D)** sex, **(E,F)** stage, **(G,H)** T stage, **(I,J)** N stage, and **(K,L)** M stage.

To value the prognostic ability of the risk signature, we compared the distribution of risk score, the pattern of survival time, the survival status, and the relevant expression of 7 NRlncRNAs between the low- and high-risk groups in the train, test, and entire sets (Figures 2A–L). These all indicated that the low-risk group had better prognoses. Meanwhile, the LUAD patients were separated into groups according to age, gender, stage, T stage, N stage, and M stage to study the relationship between the risk signature and the prognosis of LUAD patients among universal clinicopathological variables. For different classifications, except T3-4 and M1 stage (Figures 3H, L), the OS of the patients in the low-risk group was significantly longer than that of the patients in the high-risk group (Figures 3A–G, Figures 3I–K). The possible explanation of the T3–T4 and M1 stage might be the limited number of patients due to poor prognoses in advanced NSCLC. These results suggest that the predictive signature can also predict the prognosis of LUAD patients in a different group of age, gender, stage, N stage, T1-2 stage, and M0 stage.

### An Independent LUAD Prognostic Indicator of the Necroptosis-Related lncRNA Signature

**FIGURE 4:**
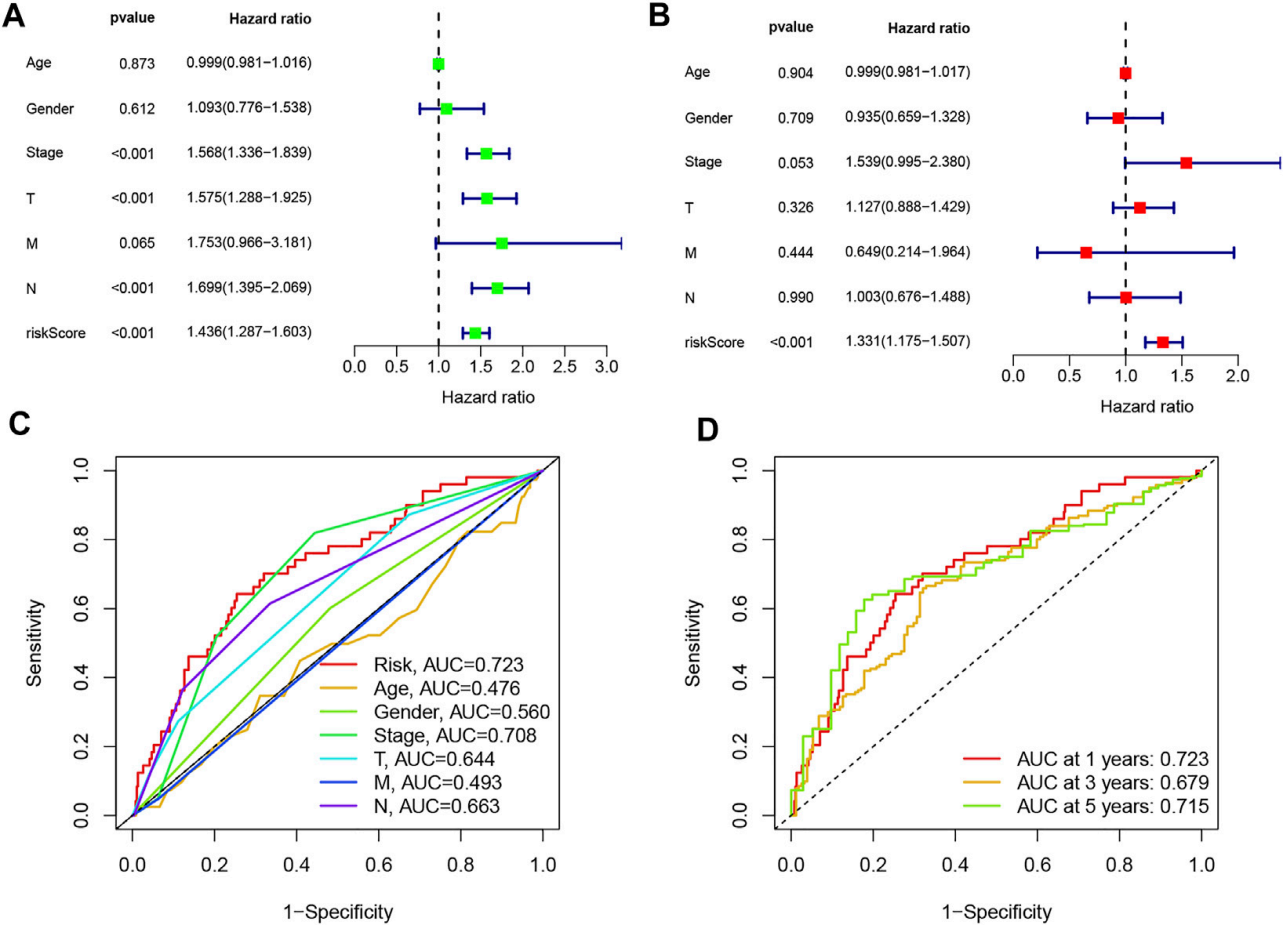
Validation of the predictive value of the prognostic signature. **(A)** Uni-Cox and **(B)** multi-Cox regression analyses of clinical characteristics and risk score with overall survival. **(C)** Comparison of the prediction accuracy of the risk model with clinicopathological features, such as age, gender, stage, T stage, M stage, and N stage. **(D)** Accuracy of the risk signature in predicting 1-, 3-, and 5-year receiver operating characteristic curves based on the entire sets.

To determine whether the predictive signature is an independent prognostic factor for LUAD patients, Cox regression analysis was performed in the entire set. The Uni-Cox regression analysis showed that stage, T stage, N stage, and risk score were significantly associated with the OS of LUAD patients (Figure 4A). The multi-Cox regression analysis showed that only risk score (hazard ratio = 1.331, confidence interval = 1.175–1.507, *p* < 0.001) was an independent predictor of OS in LUAD patients (Figure 4B). Then, we used AUC to validate the sensitivity and the specificity of the signature in the entire set. The AUC of the risk score was 0.723, which was better than that of clinicopathological variables in predicting the prognosis of LUAD patients (Figure 4C). The AUCs of 1-, 3-, and 5-year survival were 0.723, 0.679, and 0.715, respectively, which indicated a good predictive value (Figure 4D). These results further implied that the signature was a promising biomarker for indicating the prognosis risk of LUAD.

### Construction and Evaluation of the Prognostic Nomogram

**FIGURE 5:**
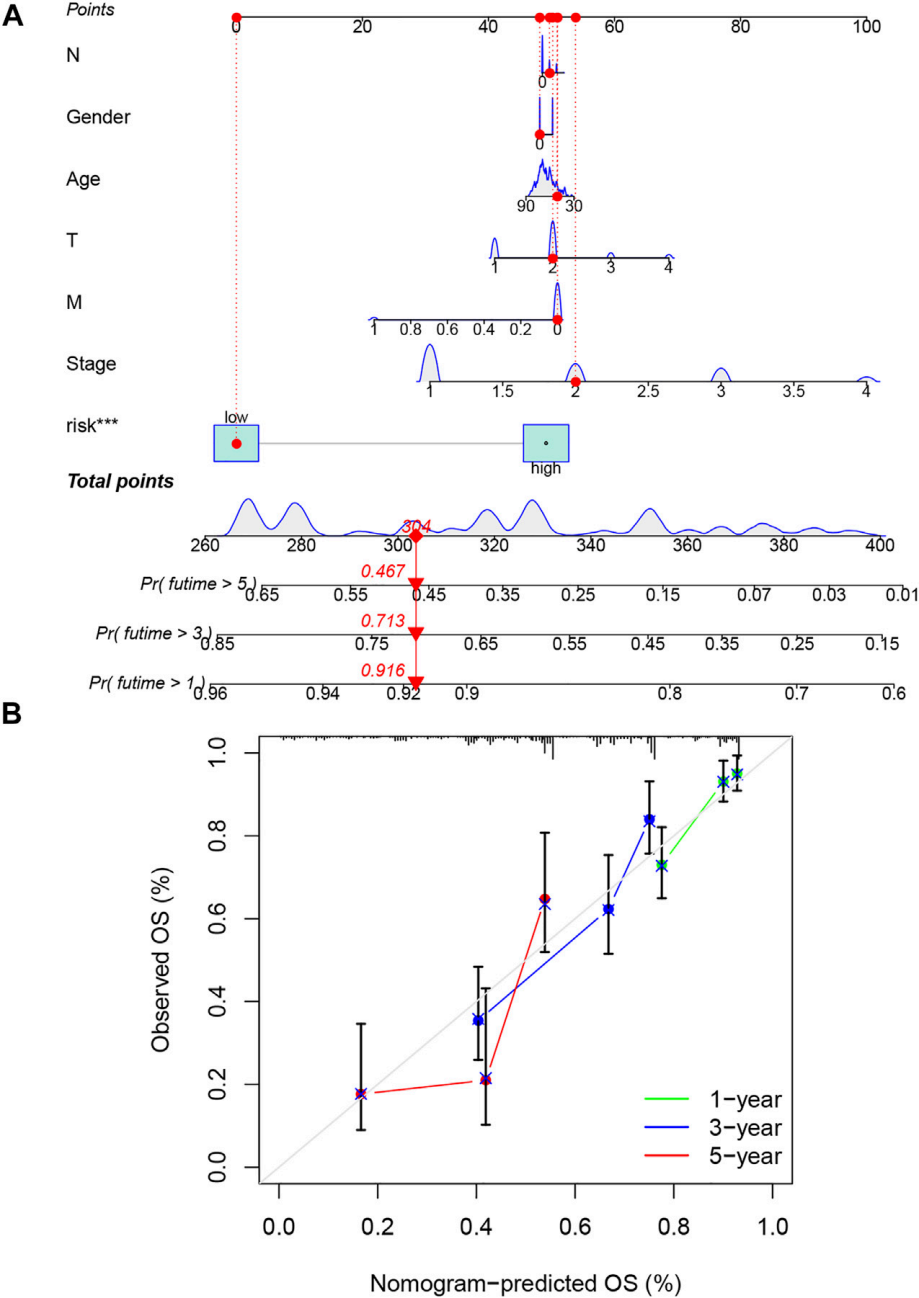
Construction and verification of the nomogram. **(A)** A nomogram combining clinicopathological variables and risk score predicts the 1-, 3-, and 5- year overall survival of lung adenocarcinoma patients. **(B)** The calibration curves test the consistency between the actual outcome and the predicted outcome at 1, 3, and 5 years.

The nomogram including clinicopathological variables and the risk score were constructed to predict the 1-, 3-, and 5-year prognosis of LUAD patients (Figure 5A). The calibration curves indicated a good consistency between the actual OS rates and the predicted survival rates at 1, 3, and 5 years (Figure 5B).

### Tumor Immune Microenvironment of the Necroptosis-Related lncRNA Signature

**FIGURE 6:**
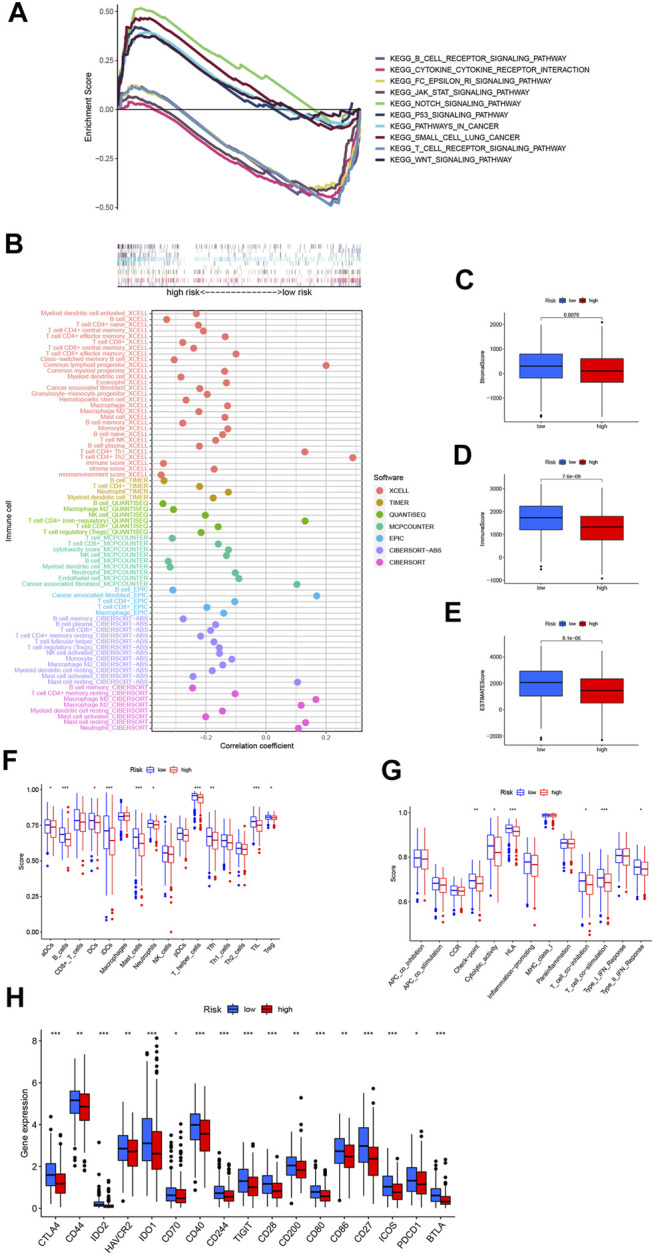
The differences of tumor immune microenvironment between the low- and high-risk groups. **(A)** Gene set enrichment analysis of the top 10 pathways significantly enriched in the risk groups. **(B)** The immune cell bubble of risk groups. **(C–E)** The box plots of the comparison of StromalScore, ImmuneScore, and ESTIMATEScore, respectively, between low- and high-risk groups. **(F, G)** The ssGSEA scores of immune cells and immune functions in the risk groups. **(H)** The difference of common immune checkpoint expression in the risk groups. *p < 0.05, **p < 0.01, ***p < 0.001.

Based on the different prognoses of patients in the high- and low-risk groups, we conducted GSEA to explore the underlying differences in biological functions between risk groups. We found that the T/B cell receptor signaling pathway, Fc epsilon RI signaling pathway, cytokine receptor interaction, and JAK-STAT signaling pathway were significantly enriched in the low-risk group (Figure 6A), indicating that low-risk patients are closely related to tumor- and immune-related pathways. The GSEA results also revealed that the Notch signaling pathway, Wnt signaling pathway, and p53 signaling pathway, pathways in cancer and small cell lung cancer, were significantly enriched in the high-risk group. The Notch pathway plays a vital role in lung tumorigenesis and progression. Researchers found that cigarette smoke could promote LUAD progression *via* activating the Notch-1 pathway (Chiappara et al., 2022). Additionally, Notch-1 signaling synergized with Hif-1α could upregulate the expression of survivin in LUAD cell line A549 (Chen et al., 2011). The overexpression of Wnt pathway-activating genes and the down-expression of negative regulators of the pathway are closely correlated with NSCLC tumorigenesis, prognosis, and resistance to therapy (Stewart, 2014; Zeybek et al., 2022). The Wnt responder cells showed an increased tumor propagation ability, suggesting that they have features of normal tissue stem cells (Tammela et al., 2017). These mechanisms may explain why the high-risk group has a worse prognosis. Then, we studied the correlation between risk scores and tumor-infiltrating immune cells (Figure 6B). More immune cells are closely related to the low-risk group on different platforms. Consistently, we also found that StromalScore, ImmuneScore, and ESTIMATEScore in low-risk patients were significantly higher than those of high-risk patients (Figures 6C–E). To further explore the correlation between risk scores and immune cells and functions, we quantified the enrichment scores of ssGSEA for different immune cell subgroups, related functions, or pathways. The results exhibited that activated dendritic cells (aDCs), B cells, DCs, immature dendritic cells (iDCs), mast cells, neutrophils, T helper cells, T follicular helper (Tfh) cells, tumor-infiltrating lymphocyte (TIL), and T regulatory cells (Tregs) were significantly negatively correlated with the risk score (Figure 6F). Compared with the high-risk group, several immune pathways, *e*.*g*., checkpoint, cytolytic activity, human leukocyte antigen (HLA), T cell co-inhibition, T cell co-stimulation, and type II IFN response were higher in the low-risk group (Figure 6G). Furthermore, by comparing immune checkpoint activation between different risk groups, we found that almost all the immune checkpoints expressed more activity in the low-risk group, such as CTLA-4, HAVCR2 (TIM3), PDCD1 (PD-1), TIGIT, and CD70 (Figure 6H). These findings suggested that, in the low-risk group, the immunological function is more active and might be more sensitive to immunotherapy.

## Discussion

As the most common subtype of lung cancer, LUAD still poses a huge threat to human health worldwide, with mounting morbidity and mortality. The identification of a specific and reliable prognostic signature for LUAD patients is extremely vital to improve the prognosis. Although there are a lot of other signatures using lncRNAs to predict the survival outcomes of LUAD, a necroptosis-related lncRNA predictive signature has not been reported. Herein we constructed a necroptosis-related lncRNA signature to explore the prognosis and TIME of LUAD patients.

In this study, 1,016 differentially expressed NRlncRNAs were acquired to explore the prognostic function. We conducted univariate, LASSO, and multivariate Cox regression analyses and identified seven NRlncRNAs (AC026355.2, AC099850.3, AF131215.5, UST-AS2, ARHGAP26-AS1, FAM83A-AS1, and AC010999.2) significantly linked to the OS of LUAD patients to construct the necroptosis-related lncRNA signature. Among those lncRNAs, AC099850.3 has been reported to be highly expressed in tumors and closely related to the development and procession of NSCLC (Zhou et al., 2021); AC099850.3 is demonstrated to promote proliferation and migration in hepatocellular carcinoma and is also an important member of the prognosis model in hepatocellular carcinoma and colorectal cancer (Wu et al., 2021; Zhang et al., 2021). AF131215.5 also represented the independent prognostic significance of OS in patients with LUAD (Hou and Yao, 2021). FAM83A-AS1 could increase FAM83A expression by enhancing FAM83A pre-mRNA stability and promote the tumorigenesis of LUAD (Wang et al., 2021). FAM83A-AS1 was also verified to contribute to LUAD proliferation and stemness *via* the HIF-1α/glycolysis axis (Chen et al., 2022). Other lncRNAs (AC026355.2, UST-AS2, ARHGAP26-AS1, and AC010999.2) were revealed for the first time. It is noteworthy that knowledge on those newly distinguished NRlncRNAs could develop a better mechanistic understanding of LUAD, which might be new targets for cancer treatment. Then, the LUAD patients were divided into high- and low-risk groups based on the median value of the risk score. The results all indicated that the low-risk group had a better prognosis than the high-risk group, and risk score was an independent predictor of OS in LUAD patients. The ROC analysis showed that the signature was superior to conventional clinical characteristics in the survival prediction of LUAD. Similarly, the predictive nomogram established also showed a perfect consistency between the observed and predicted rates for the 1-, 3-, and 5-year OS. Collectively, these studies mentioned above indicate that our necroptosis-related lncRNA signature could predict the prognosis of LUAD patients accurately.

Researchers have demonstrated that necroptosis is strongly associated with tumorigenesis, tumor immune response, and poor prognosis (Gong et al., 2019), especially in solid tumors, but the specific role of necroptosis in those processes is still largely unknown. Therefore, we continued to explore the underlying mechanism of necroptosis-related lncRNA signature among different risk groups.

GSEA showed that the T/B cell receptor signaling pathway, Fc epsilon RI signaling pathway, cytokine receptor interaction, and JAK/STAT signaling pathway were significantly enriched in the low-risk group. Researchers found that the aberrant activation of the JAK/STAT signaling pathway was closely related to the occurrence, development, metastasis, and drug resistance of lung cancer (Li S. D. et al., 2017). The overexpression of JAK2 induced the proliferation, migration, and invasion abilities of lung adenocarcinoma A549 cells; conversely, the downregulation of JAK2 could suppress the protumorigenic effect (Xu et al., 2017). EGFR tyrosine kinase inhibitors (TKIs), such as afatinib and dacomitinib, could activate STAT3 *via* autocrine interleukin-6 (IL-6) production, and that blockade of the IL-6R/JAK1/STAT3 signaling pathway potentiated sensitivity to those EGFR TKIs in NSCLC cells (Kim et al., 2012). In addition, the researcher found that zVAD (a pan-caspase inhibitor) induced necroptotic death in TLR3- and TLR4-activated macrophages *via* the JAK/STAT1/ROS pathway (Chen Y. S. et al., 2021). IFN-activated JAK/STAT signaling induced the robust expression of ZBP1, which complexed with RIPK3 to trigger MLKL-driven necroptosis (Ingram et al., 2019). Similarly, TNF-α synergized with IFN-γ could induce epithelial cell necroptosis through the CASP8-JAK1/2-STAT1 module (Woznicki et al., 2021). Taken together, we speculated that necroptosis probably contributed to the occurrence and development of LUAD through the JAK/STAT signaling pathway.

According to the role of necroptosis in regulating tumor immunity and the enrichment of immune-related pathways in low-risk groups, we performed ssGSEA to explore the immune status in different groups. The immune cells (aDCs, B cells, DCs, iDCs, mast cells, neutrophils, T helper cells, Tfh cells, TIL, and Tregs) and immune functions (checkpoint, cytolytic activity, HLA, T cell co-inhibition, T cell co-stimulation, and type II IFN response) were mainly active among the low-risk groups, some of which were closely linked to necroptosis. Necroptotic cells can provide both tumor-specific antigens and inflammatory cytokines to DCs for antigen cross-priming which activates cytotoxic CD8^+^ T lymphocytes. RIPK3 was necessary for the regulation of cytokine expression in DCs, which could participate in innate and adaptive immune systems (Park et al., 2021). Wang X *et al*. found that a serine protease was involved in the RIPK3–MLKL-mediated necroptotic death pathway in neutrophils (Wang X et al., 2020). These results further illustrated that necroptosis might be involved in the progression of LUAD by regulating tumor immunity. Subsequently, we analyzed the correlation between common immune checkpoint expression and necroptosis-related lncRNA signature. Some researchers have indicated that the expression levels of immune checkpoint genes are highly associated with the efficacy of immunotherapy (Ahluwalia et al., 2021; Hu et al., 2021). Our findings demonstrated that most of the immune checkpoints’ expression was elevated in low-risk LUAD patients compared to the high-risk group. Among those, PD-1 and CTLA-4 inhibitors have been validated to benefit patients with advanced NSCLC in clinical trials (Paz-Ares et al., 2021). In addition, TIM3, TIGIT, and CD70 have been under investigation, and drugs blocking these immune checkpoints are in clinical or preclinical developments (Bewersdorf et al., 2021; Hansen et al., 2021). Therefore, this signature implied that it would be more advantageous for LUAD patients at a lower risk to receive immunotherapy.

However, our research has several limitations and shortcomings. Firstly, it was better to include more clinical databases for external validation. Secondly, the underlying molecular mechanisms of the NRlncRNAs in LUAD should be further validated by experiments. Thus, we will recollect and expand clinical samples and attempt to validate the accuracy of this model *via* more external experiments in our following work.

In conclusion, the necroptosis-related lncRNA predictive signature can independently predict the prognosis of LUAD patients, helps elucidate the process and mechanism of NRlncRNAs in LUAD, and provides immunotherapy guidance for patients with LUAD, but it still needs further experimental verification in the future.

## Data Availability

The original contributions presented in the study are included in the article/Supplementary Material, further inquiries can be directed to the corresponding author.

## Abbreviations

AUC, area under the ROC curve; GSEA, gene set enrichment analysis; GSVA, gene set variation analysis; LASSO, least absolute shrinkage and selection operator; lncRNA, long non-coding RNA; LUAD, lung adenocarcinoma; NRlncRNAs, necroptosis-related lncRNAs; NSCLC, non-small cell lung cancer; OS, overall survival; ROC, receiver operating characteristic curve; ssGSEA, single-sample gene set enrichment analysis; TCGA, the cancer genome atlas; TIME, tumor immune microenvironment.
