# Supplementary material for: A Novel Necroptosis-Related lncRNA Signature Predicts the Prognosis of Lung Adenocarcinoma
Source: Front Genet. 2022 Mar 17;13:862741. doi: 10.3389/fgene.2022.862741 (PMC8969905; doi:10.3389/fgene.2022.862741)
Supplement: Supplementary file 1 [file DataSheet1.PDF]

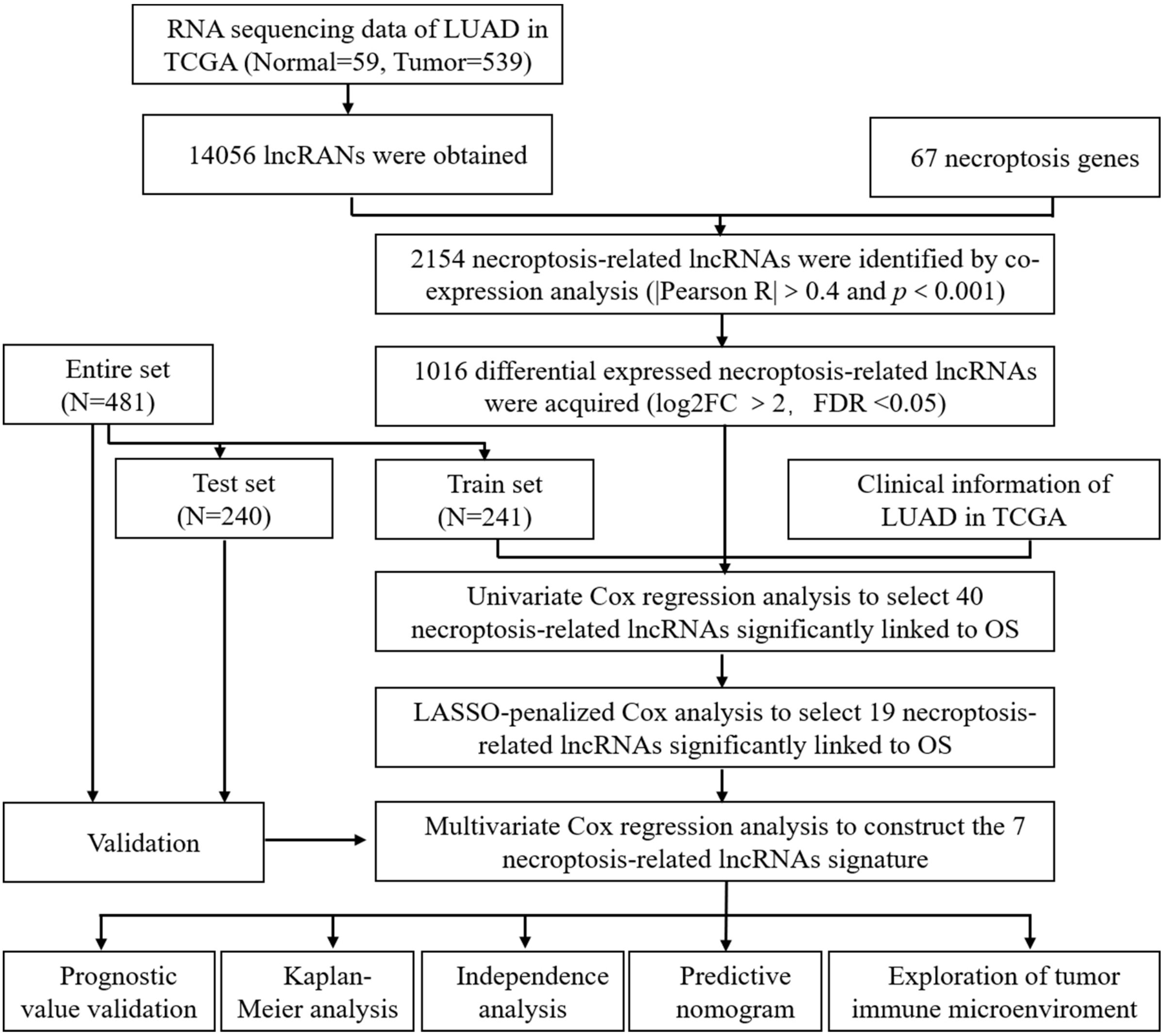

Supplementary Figure S1. The schematic workflow of this study.

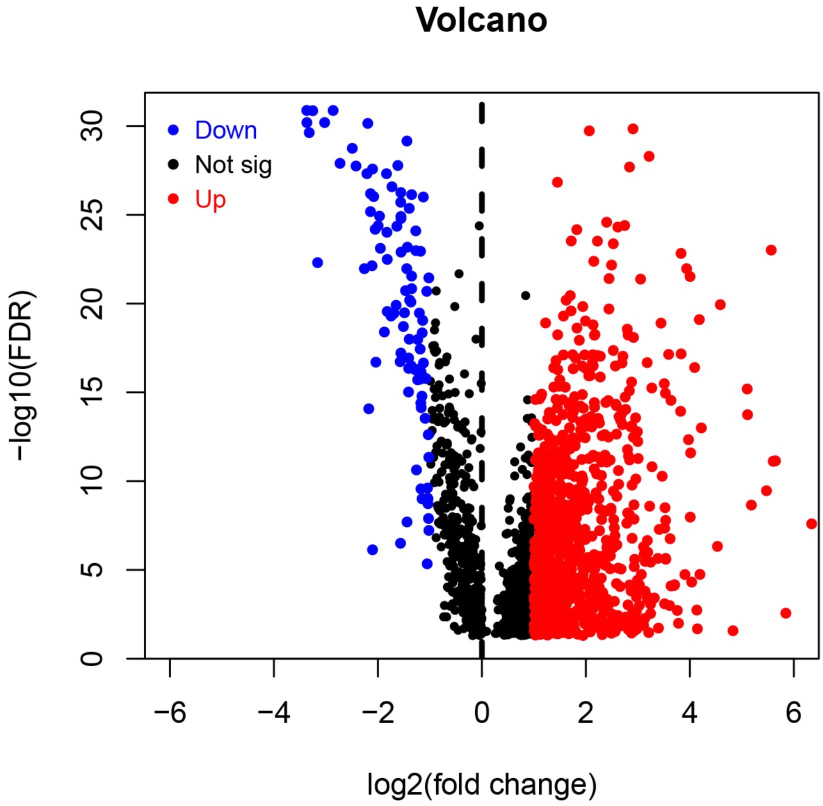

Supplementary Figure S2. The volcano plot of 1016 differentially expressed necroptosis-related lncRNAs (red, black, and blue represents Up, Not, and Down significant).
